# Are Plasma Oxytocin and Vasopressin Levels Reflective of Amygdala Activation during the Processing of Negative Emotions? A Preliminary Study

**DOI:** 10.3389/fpsyg.2016.00480

**Published:** 2016-04-08

**Authors:** Kosuke Motoki, Motoaki Sugiura, Hikaru Takeuchi, Yuka Kotozaki, Seishu Nakagawa, Ryoichi Yokoyama, Ryuta Kawashima

**Affiliations:** ^1^Institute of Development, Aging and Cancer, Tohoku UniversitySendai, Japan; ^2^International Research Institute of Disaster Science, Tohoku UniversitySendai, Japan; ^3^Medical-Industry Translational Research Center, Fukushima Medical University School of MedicineFukushima, Japan; ^4^Department of Psychiatry, Tohoku Pharmaceutical UniversitySendai, Japan; ^5^Japan Society for the Promotion of ScienceTokyo, Japan

**Keywords:** oxytocin, vasopressin, emotional processing, sex differences, functional magnetic resonance imaging

## Abstract

Plasma oxytocin (OT) and arginine vasopressin (AVP) are associated with individual differences in emotional responses and behaviors. The amygdala is considered to be an important brain region for regulating emotion-based behavior, with OT and AVP modulating activity in the amygdala during the processing of negative emotions. In particular, increased OT levels may diminish amygdala activation (anxiolytic effects) and enhanced AVP levels may augment amygdala activation (anxiogenic effects) when negative emotions are processed. A growing body of research has shown that the effects of OT and AVP are modulated by sex: the aforementioned anxiolytic effects of OT and the anxiogenic effects of AVP occur in men, but not in women. However, we have little knowledge regarding the biological mechanisms underlying OT and AVP plasma levels or their respective anxiogenic and anxiolytic effects; similarly, little is known about the causes and nature of sex differences related to these neuropeptides and their effects on emotional processing. In the current study, we focused on the neural functions associated with the biological mechanisms underlying such effects. We hypothesized that amygdala activation would correlate with trait plasma OT (anxiolytic effects) and AVP (anxiogenic effects) levels because the amygdala is thought to affect the coordinated release of these neuropeptides following affective experiences. We further hypothesized that the effects would be modulated by sex. We assessed 51 participants (male and female) using a paradigm involving negative emotion in conjunction with functional magnetic resonance imaging and measurements of plasma OT and AVP levels. We determined that increased plasma AVP levels were positively associated with amygdala activation (anxiogenic effects) in men, but not in women. These findings highlight the potential underlying neural mechanisms of plasma AVP levels in men.

## Introduction

Emotions are basic human biological features; to survive in everyday life requires the ability to monitor, evaluate, and modify our emotional states, and to adaptively express our emotions. However, not all humans process their emotional experiences in the same manner, with individual differences arising in the evaluation and expression of emotions upon encountering affect-inducing experiences. These individual differences are known to depend on personality traits, cognitive factors, and situational influences (Buchanan et al., 1999; Kim et al., 2004; Drabant et al., 2009; Somerville et al., 2013). In addition, neuropeptides (e.g., as manifested in their central and plasma concentrations) modulate individual responses to affective stimuli (Holmes et al., 2003). Despite the known importance of neuropeptides with respect to emotional responses, the mechanisms that govern individual differences and the means by which they modulate neural responses to emotional experiences have not been fully elucidated.

Among the various neuropeptides, the affective functions of oxytocin (OT) and arginine vasopressin (AVP) in particular have increasingly drawn the attention of researchers (Caldwell et al., 2008; Raggenbass, 2008; Meyer-Lindenberg et al., 2011). While OT is involved in sexual reproduction, and AVP affects water retention and vasoconstriction, individual differences in plasma OT and AVP are also implicated in emotional behaviors and affective disorders, though there is considerable controversy regarding the relationship between peripheral levels of these neuropeptides and their central availability (Meyer-Lindenberg et al., 2011). For example, their concentrations in cerebrospinal fluid (CSF), a global measure of the central release of neuropeptides, are positively correlated with plasma levels in some studies (Bartrons et al., 1992), but not in others (Wu and Keysar, 2007).

Although there is still controversy regarding the relationship between peripheral levels of these neuropeptides and their central availability, numerous studies have used plasma OT and AVP shows the associations between emotional variables and plasma OT and AVP, which implies that their plasma levels reflect their central levels. For example, lower plasma OT levels and higher AVP levels have been linked with several psychiatric disorders, including major depressive disorders (Londen et al., 1997; de Winter et al., 2003) and posttraumatic stress disorder (De Kloet et al., 2008), that have been characterized as involving increased amygdala activation (Siegle et al., 2002; Etkin and Wager, 2007). Hence, the augmented activation of the amygdala during emotional processing that is present in these psychiatric disorders may be due to decreased OT and increased AVP levels. The above findings led us to view OT as potentially exerting anxiolytic effects (i.e., decreased amygdala activation for negative emotions) and AVP as inducing anxiogenic effects (i.e., augmented amygdala activation for negative emotion).

It is known that sex modulates the anxiolytic effects of OT and the anxiogenic effects of AVP. Evidence has accumulated indicating that AVP exerts anxiogenic effects in men but not in women. For example, higher AVP levels, which may be linked with augmented amygdala activation during emotional processing, have been associated with an increase in traits related to negative affect in men, but not in women (Taylor et al., 2010). Furthermore, AVP administration has been shown to increase amygdala activation in male subjects perceiving fearful stimuli (Brunnlieb et al., 2013; Lee et al., 2013). In contrast, the anxiolytic effects of OT are more complex and may exert an opposite sex-based difference. Notably, intranasally administered OT was found to *decrease* amygdala activation in men (Kirsch et al., 2005; Domes et al., 2007; Petrovic et al., 2008; Labuschagne et al., 2010; Riem et al., 2011), but increase it in women (Domes et al., 2007; Lischke et al., 2012). In addition, plasma OT is negatively correlated with trait anxiety, which is related to increased amygdala activation during emotional processing (Stein et al., 2007) in men but not in women (Weisman et al., 2013). These findings suggest that the anxiogenic effects of AVP and the anxiolytic effects of OT appear in men but not in women.

As indicated in the above discussion, numerous studies have used plasma OT and AVP as a measure of individual differences pertinent to the anxiolytic effects of OT and the anxiogenic effects of AVP, assuming that their plasma levels reflect their central levels. However, the underlying biological mechanisms of the anxiogenic effects of AVP and the anxiolytic effects of OT remain unclear Accordingly, the present study aimed to investigate the biological mechanisms underlying the relationships between plasma levels of OT and AVP and their anxiogenic and anxiolytic effects, and how sex differences may modulate these effects.

We hypothesized that the amygdala would be a key region for investigating the underlying biological functions of individual differences pertaining to the anxiolytic effects of OT and the anxiogenic effects of AVP, with anxiolytic effects manifesting as decreased amygdala activation correlated with OT, and anxiogenic effects as increased amygdala activation correlated with AVP during the processing of negative emotion. First, the anxiolytic effects of OT and the anxiogenic effects of AVP may be mediated by receptors in the amygdala (Meyer-Lindenberg et al., 2011). Although receptors for OT and AVP are widely expressed in the hypothalamus and the bed nucleus of the stria terminalis as well as in the amygdala (Loup et al., 1991; Veinante and Freund-Mercier, 1997; Young et al., 1999), among these, the amygdala is critically involved in the processing of negative emotions (Öhman, 2005). Injections of OT or AVP in the amygdala were found to decrease or increase, respectively, fearful behaviors related to depressive symptoms in animals (Huber et al., 2005; Salome et al., 2006). All of this evidence suggests that activity in the amygdala is related to plasma levels of OT and AVP during aversive experiences. Second, the amygdala may be a potential target for communication between central and peripheral OT and AVP release following negative affective responses, and as a result, baseline levels of OT and AVP may partially reflect their central levels in the amygdala; however, the details of the potential mechanisms remain unclear (McCullough et al., 2013). An experimental model of peripheral and central communication with respect to these neuropeptides suggests that following a response to an affective stimulus, OT and AVP are released in the amygdala because this region contains high densities of OT and AVP neurons and receptors. Subsequently, affective OT- and AVP-derived responses in the amygdala are transmitted to other limbic regions, leading to the projection of magnocellular hypothalamic OT and AVP neurons to the posterior pituitary. Finally, these neuropeptides are conveyed to peripheral routes through the posterior pituitary (Neumann and Landgraf, 2012). However, there is no evidence to determine whether amygdala activation during the processing of negative emotion is associated with individual differences in OT and AVP plasma levels.

This study aimed to investigate the biological mechanisms underlying the anxiolytic effects of OT and the anxiogenic effects of AVP related to the peripheral levels of these neuropeptides and to identify any sex-based modulation. To this end, we investigated the potential association between plasma OT and AVP levels and amygdala activation during the processing of negative emotions in both men and women. To conduct our investigation, we used functional magnetic resonance imaging (fMRI) and a negative emotion paradigm known to robustly induce amygdala activation (Ochsner et al., 2009; Silvers et al., 2015). We hypothesized that: (1) decreased amygdala activation during the processing of negative emotion (anxiolytic effects) would be associated with elevated plasma OT levels in men but not in women; and (2) that increased amygdala activation during negative emotional responses (anxiogenic effects) would be associated with elevated plasma AVP levels in men but not in women.

## Methods

### Participants

A total of 73 healthy, right-handed volunteers with no history of neurological or psychiatric illness participated in our fMRI study. Participants were recruited through notices on the university campus and announcements in local newspapers. They provided written informed consent after receiving an explanation regarding the nature of the experiment. This study was performed concomitantly with another intervention study investigating the effects of training on shadowing, compressed speech, and reading aloud. These studies shared a pool of active control group subjects, psychological and neuroimaging outcome measures, a training period, training times, and training frequencies. All procedures were conducted according to the Declaration of Helsinki. The experimental protocol was approved by the ethics committee of the Tohoku University School of Medicine.

We removed participants who did not perform the fMRI task or who exhibited excessive bodily movements during MRI (*n* = 4), as well as those whose plasma level of either OT or AVP was below the detection threshold or missing a value (*n* = 17). We then converted plasma OT and AVP to log values, and subjects with OT or AVP values that exceeded three standard deviations (SD) were removed (*n* = 1). We ultimately used data from a total of 51 subjects (age: 18–26 years; 27 males). The mean age was in 20.63 ± 1.690 for men, and 20.17 ± 0.917 for women. The mean OT value was 20.9392 pg/mL ± 5.93579 for all participants, 21.7667 pg/mL ± 7.25253 for men, and 20.0083 pg/mL ± 3.92771 for women. The mean AVP value was 1.874510 pg/mL ± 1.1014251 for all participants, 2.1148 pg/mL ± 1.29011 for men, and 1.6042 pg/mL ± 0.78213 for women. Data pertaining to the women's menstrual cycles on the day of blood sampling was available for 23 of the 24 women (menstrual phase: *n* = 5; luteal phase: *n* = 6; follicular phase: *n* = 12). These menstrual cycle stages were categorically coded for further analysis (menstrual phase = 0; luteal phase = 1; follicular phase = 2). None of the women were taking oral contraceptives.

### Assessment of chronic negative affect

Trait anxiety levels were assessed using the Japanese version of the State-Trait Anxiety Inventory (STAI) (Spielberger, 2010). Data were available for 49 of the 51 participants.

### Laboratory trait plasma OT and AVP biomarkers

To capture baseline (i.e., trait) levels of plasma OT and AVP, we obtained heparinized blood samples on a single day from the antecubital vein of subjects after they had rested for 15 min. Sampling occurred within 1 week of the MRI experiment and blood was obtained between 2:00 and 5:00 p.m. to minimize diurnal variation among subjects. The blood samples were centrifuged at 2000 × g at 4°C for 25 min, and the plasma was subsequently harvested and frozen at −80°C until further investigation. We assessed plasma OT levels using a commercially available enzyme immunoassay kit with a detection limit of (Enzo Life Sciences, Inc., Farmingdale, NY, USA). Validation of this assay procedure has been described (Sue Carter et al., 2007) and repeatedly conducted in previous studies investigating plasma OT in humans (Feldman et al., 2007, 2010). All sample extraction and concentration procedures were conducted at the LSI Medicine Corporation (Sendai, Japan) in accordance with the official kit manual. Although OT levels fluctuate between the time of blood sampling and brain scanning (especially in females), our assay indicated a reliable stability from day-to-day. According to the assay's protocol, the inter-assay coefficients of variability are lower than 20.9% and the intra-assay coefficients are lower than 13.3% (Enzo Life Sciences, Inc.). The assay also refers to the stability of inter- and intra-assay coefficients of variability observed in a previous study (Lancaster et al., 2015). A natural log transformation was applied to correct any skewing in plasma OT values.

Plasma AVP levels were similarly measured according to the manufacturer's instructions, using an AVP radioimmunoassay (RIA) Neo double antibody method (LSI Medicine Corporation) with a detection limit of 0.8 pg/mL. This assay was used in a prior study (Imamura et al., 2014). Although AVP levels fluctuate between during the time of blood sampling and brain scanning (especially in females), our assay indicated the reliable stability from day-to-day. According to the manufacturer's protocol, the inter- and intra-assay coefficients of variability are less than 10%, indicating remarkable stability of the samples and reliability of the assay (LSI Medicine Corporation). A natural log transformation was applied to correct skewing of the plasma AVP values.

### fMRI stimuli

A total of 26 negative and 13 neutral pictures, according to normative ratings, were selected from the International Affective Picture System (IAPS). On a 9-point scale, the mean valence and arousal values for the neutral pictures were 5.149 ± 0.191 and 3.401 ± 0.459, respectively, and for the negative pictures, 2.067 ± 0.387 and 6.157 ± 0.606, respectively (1: most unpleasant/least arousing; 9: most pleasant/most arousing) (Lang et al., 1997).

### Emotional reactivity task

The emotional reactivity task was conducted as described in previous studies (Ochsner et al., 2009; Wager et al., 2013). This task consists of three conditions, Look Neutral (viewing of a neutral picture), Look Negative (viewing of a negative picture), and Decrease Negative (reinterpretation of a negative picture), and permits contrasts related to blood oxygen level-dependent (BOLD) signal changes evoked by naturally occurring emotional reactions to negative stimuli (Look Negative vs. Look Neutral). We focused on the Look Neutral and Look Negative conditions for our objective evaluation of emotional reactivity. The Decrease Negative condition was used in our other study, which ran concomitantly with the current one; its purpose was unrelated to the present research. For the Look Neutral and Look Negative conditions, participants were shown either neutral or aversive images and were instructed to react naturally to the stimuli (i.e., without reappraising them). In contrast, in the Decrease Negative condition, subjects were instructed to reappraise the images in a way that reduced their negative impact.

Each trial proceeded as follows. First, an instruction (“Look” or “Decrease”) appeared on the screen for 2 s (as indicated above, the results following the instruction to “Decrease” will be detailed elsewhere because this instruction aimed to investigate emotional regulation, which is outside the scope of the present study). Subsequently, a neutral or negative image was presented for 7 s. A fixation cross then appeared for a variable interstimulus interval (1–3 s), followed by an emotional rating phase of 2 s in duration. During the rating phase, participants were asked to rate the strength of their negative feelings on a scale of 1–8 (1 = not negative at all, 8 = very negative), which appeared from left to right on the screen. The directionality of the affective rating scale was counter-balanced so that half the trials used a reversed scale with 8 on the left and 1 on the right side of the screen; this was done to prevent hand movement preparation during exposure to the emotional picture. After the emotional rating phase, the trial concluded with a fixation cross that appeared for a variable duration of 1–3 s.

Thirteen negative images were preceded by the “Look” instruction, and 13 negative images were preceded by the “Decrease” instruction. An additional 13 neutral images were preceded by only the “Look” instruction because no emotional regulation is required for neutral images. The three conditions (Look Neutral, Look Negative, and Decrease Negative) were randomly intermixed throughout the experiment to avoid habituation to the same type of instructions or images appearing consecutively. Prior to scanning, participants received an explanation of reappraisal and reactivity strategies. During this time, participants were also reminded not to look away from the images or distract themselves with irrelevant and/or positive thoughts unrelated to the images. After all of the instructions had been conveyed, participants received a brief training session regarding emotional reactivity and regulation. The images used during training were different from those used during the subsequent test.

### Behavioral analyses

To determine the relationships between plasma OT and AVP levels and subjective reports of negative emotions during the emotional reactivity task, we performed multiple regression analyses using SPSS (version 21; IBM, Armonk, NY, USA). We used subjective scores of negative emotion as predictors. First, using OT (or AVP), age, and sex as explanatory variables, we analyzed the relationship between OT (or AVP) and negative emotion scores. Second, using the OT-by-sex (or AVP-by-sex) interaction term, OT (or AVP), age, and sex (coded as men = 1, women = −1) as explanatory variables, we analyzed the relationship between OT-by-sex (or AVP-by-sex) and negative emotion scores. OT, AVP, sex, and their interaction terms were centered in the second analysis to avoid multicollinearity. Also, menstrual cycle stages were categorically coded and entered into multiple regression analyses for no-interests of explanatory variables (menstrual phase = 0; luteal phase = 1; follicular phase = 2). The analyses were performed using SPSS (version 22). All tests were two-tailed, with a threshold of *p* < 0.05.

### fMRI methods and data acquisition

For all sessions, 42 transaxial gradient-echo images (echo time = 10 ms, flip angle = 90°, slice thickness = 3 mm, slice gap = 0 mm, field of view [FOV] = 192 mm, matrix = 64 × 64, voxel size = 3 × 3 × 3 mm) covering the entire cerebrum were acquired at a repetition time of 2.5 s using an echo planar sequence and an Achieva (3T) MR scanner (Philips, Amsterdam, Netherlands).

The following preprocessing procedures and subsequent statistical analyses were performed using Statistical Parametric Mapping (SPM8) software (Wellcome Department of Imaging Neuroscience, London, UK) and MATLAB®: adjustment of acquisition timing across slices, correction for head motion, spatial normalization using the EPI-MNI template, and smoothing using a Gaussian kernel with a full width at half-maximum of 8 mm. The normalization step yielded BOLD images at 3 × 3 × 3 mm voxels.

All subjects who participated in this study also participated in the aforementioned additional intervention studies, and the MRI scans not reported in the present study were performed together with those described herein. Other scans included diffusion-weighted imaging, resting state fMRI, high-resolution T1-weighted structural imaging, and another fMRI task (N-back).

### fMRI analyses

A conventional two-level approach using SPM8 was adopted for event-related fMRI data (Friston et al., 1998). A voxel-by-voxel multiple regression analysis was conducted for the first-level within-subject (fixed effects) model. Expected signal changes were modeled using rectangular waveforms, convolved with the default SPM hemodynamic response functions for the three conditions (Look Neutral, Look Negative, and Decrease Negative). Low-frequency BOLD signal noise was removed by high-pass filtering (128 s cut-off).

To evaluate our hypotheses, we first conducted statistical analyses pertaining to the emotional reactivity contrast (Look Negative-Look Neutral) associated with the parameter estimates of the second-level between-subject (random effects) model using one-sample *t*-tests. The statistical threshold was set to *p* < 0.001 for height and corrected to *p* < 0.05 for multiple comparisons (false discovery rate) using cluster size and assuming the whole brain as the search volume (Genovese et al., 2002). We defined the functional regions of interest (ROIs) within brain regions that encompassed voxels above our significance threshold during the emotional reactivity task (Labuschagne et al., 2010). Using these functional ROIs, we evaluated our primary hypothesis regarding the relationship between individual differences in OT and AVP levels and brain activity associated with emotional reactivity. MarsBaR was used to extract the average parameter estimates within each functional ROI regarding contrasts in emotional reactivity on an individual basis.

Next, multiple regression analyses were performed to test the relationships between neuropeptide (OT and AVP) levels and brain activation during the processing of negative emotions, with brain activation used as a predictor. In our first regression analysis, using OT (or AVP), age, and sex as explanatory variables, we analyzed the relationship between OT (or AVP) and brain activation. In our second regression analysis, using the OT-by-sex (or AVP-by-sex) interaction term, OT (or AVP), age, and sex (coded men = 1, women = −1) as explanatory variables, we analyzed the relationship between OT-by-sex (or AVP-by-sex) and brain activation. OT, AVP, sex, and their interaction terms were centered in the second analyses to avoid multicollinearity. Also, menstrual cycle stages were categorically coded and entered into multiple regression analyses for no-interests of explanatory variables (menstrual phase = 0; luteal phase = 1; follicular phase = 2). In the third analysis, using trait anxiety and trait anxiety-sex interaction, age, and sex, as explanatory variables, we analyzed the relationship between trait anxiety, trait anxiety-sex interaction, and brain activation. Trait anxiety and interaction terms were also centered.

The analyses were performed using SPSS (version 22). All tests were two-tailed with a threshold of *p* < 0.05.

## Results

### Subjective emotional reports of negative emotion

Analyses of emotional reports from emotional reactivity trials revealed that negative images evoked stronger negative affect than did neutral images, according to self-reported scores on an 8-point scale (mean ± standard error [SE], 6.429 ± 0.698 vs. 1.567 ± 0.896, respectively; paired *t* = 22.063, *p* < 0.001).

### Trait OT, self-reports of negative emotion, and sex differences

OT did not contribute uniquely to amygdala activation during emotional reactivity trials (β = −0.117, *t* = −0.760, *p* = 0.451). However, the interaction term between OT and sex exhibited a significant independent effect, indicating that OT levels negatively correlated with self-report of negative emotion only in women [β = 0.371, *R*^2^ Change = 0.094, *F* Change (50) = 5.579, *p* = 0.022]. However, OT did not contribute to self-reported negative emotion in women after controlling for the stage in the menstrual cycle (β = −0.418, *t* = −2.039, *p* = 0.056).

### Trait AVP, self-reports of negative emotion, and sex differences

No significant association was observed between AVP and subjective reports of negative emotion (β = 0.110, *t* = 0.775, *p* = 0.442) Furthermore, there was no significant association between AVP-by-sex interaction and subjective emotional reports (β = 0.125, *t* = 0.890, *p* = 0.378). AVP did not contribute to negative emotion in women (β = −0.091, *t* = −0.413, *p* = 0.684) once the stage in the menstrual cycle was controlled for.

### Emotional reactivity-related activation

**Figure 1:**
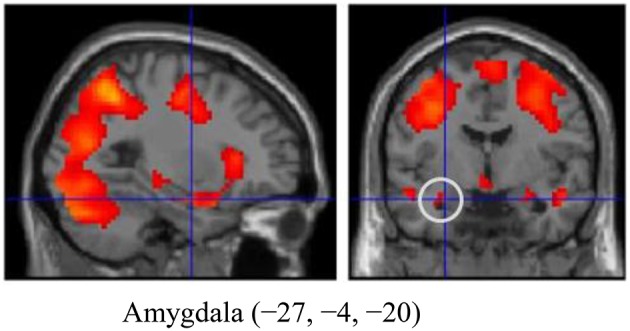
**The amygdala region (MNI coordinates: −27, −4, −20) during emotion generation (Look Negative - Look Neutral), with the statistical threshold set to *p* < 0.001 for height, corrected to *p* < 0.05 for multiple comparisons according to cluster size and assuming the whole brain as a search volume**. In this figure, activation is presented at a threshold of *p* = 0.05 for illustration purposes only. Activation is shown on an SPM8 canonical T1 image.

The emotional reactivity trial results (Look Negative-Look Neutral) are shown as Supplementary Data in Table S1 and Figure S1. We observed increased activation in the amygdala (Figure 1), the medial frontal gyrus, the inferior frontal gyrus, and in other regions. We further defined these regions as functional ROIs to analyze the relationship between OT (or AVP) and brain responses to emotional reactivity. The values for Cohen's d and effect size related to amygdala activation were 0.888 and 0.406, respectively.

### The relationship between trait OT and amygdala activation during the processing of negative emotion

Neither OT nor the OT-by-sex interaction significantly affected the amygdala (β = −0.257, *t* = −1.584, *p* = 0.129; β = 0.234, *t* = 1.384, *p* = 0.173, respectively) or other brain regions. After correction for stage in the menstrual cycle, OT did not show significant effects on amygdala activation in women (β = −0.587, *t* = −0.347, *p* = 0.132).

### The relationship between trait AVP and amygdala activation during the processing of negative emotion

**Figure 2:**
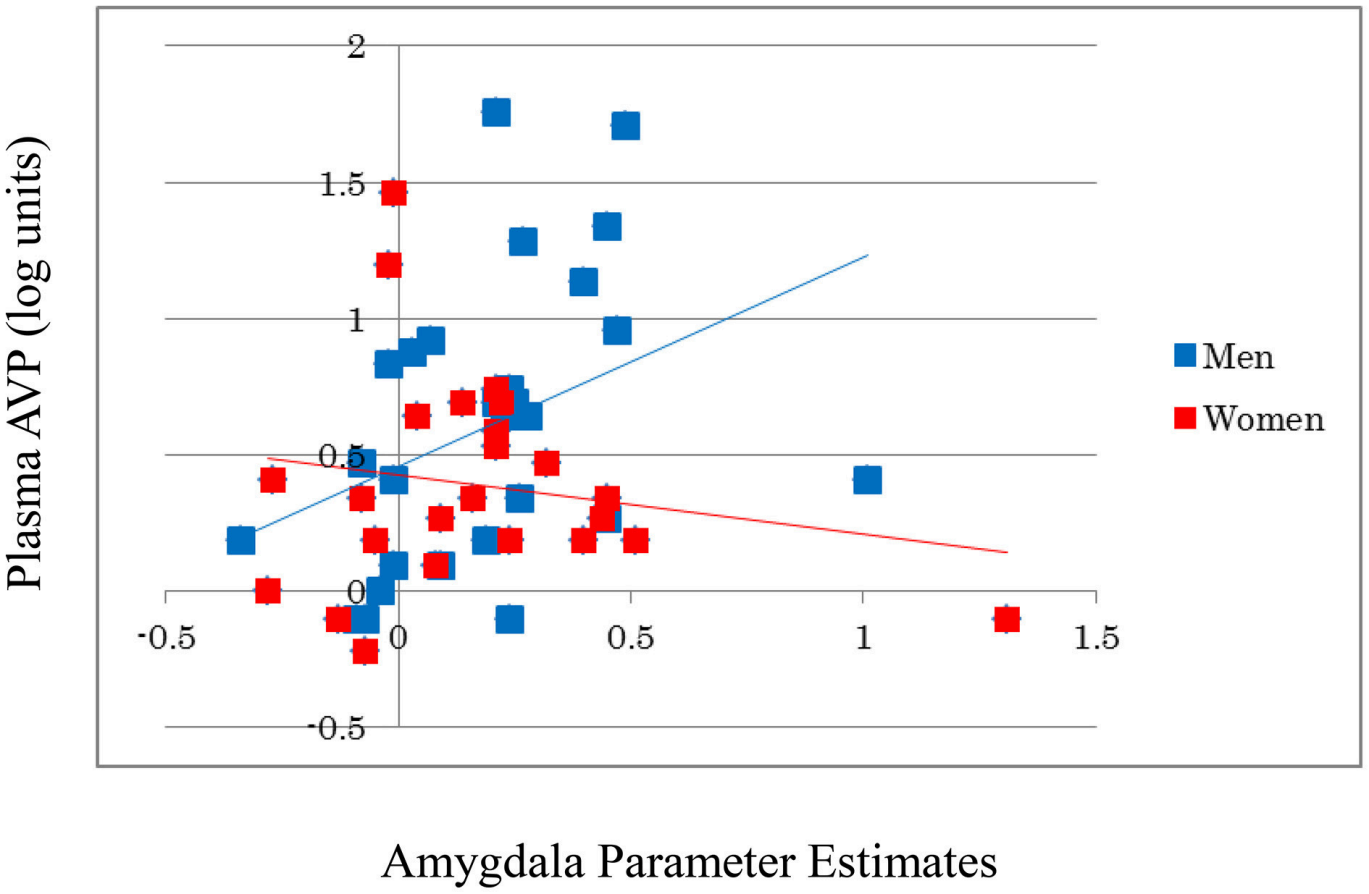
**A significant positive correlation is observed between the log-transformed plasma AVP values and amygdala activity during emotion generation (Look Negative–Look Neutral) in men, but not in women**. For illustration purposes, scatter plots are unadjusted for any covariates.

AVP did not significantly affect activation in the amygdala (β = 0.029, *t* = 0.199, *p* = 0.843) or in other brain regions during the emotional reactivity trials. However, the AVP-by-sex interaction significantly affected the amygdala [β = 0.293, *R*^2^ Change = 0.080, *F* Change (50) = 4.232, *p* = 0.045], but not other regions, indicating that trait plasma AVP may play a substantial role in emotional processing in men, but not women (Figure 2). After correction for stage in the menstrual cycle, AVP did not show significant effects on amygdala activation in women (β = −0.128, *t* = −0.562, *p* = 0.581).

### The relationship between trait anxiety and amygdala activation during the processing of negative emotion

Neither trait anxiety nor trait anxiety-by-sex interaction significantly affected the amygdala (β = −0.161, *t* = −0.948, *p* = 0.348; β = 0.062, *t* = 0.386, *p* = 0.701, respectively) or other brain regions. After correction for stage in the menstrual cycle, trait anxiety did not show significant effects on amygdala activation in women (β = −0.206, *t* = −0.914, *p* = 0.372).

## Discussion

The present study investigated the neural mechanisms of the anxiolytic effects of OT and the anxiogenic effects of AVP in plasma as well as the sex-based modulation of such effects, focusing on the amygdala. Although a growing body of research has provided empirical support for the anxiolytic effects of OT and the anxiogenic effects of AVP in plasma (Londen et al., 1997; De Kloet et al., 2008; Taylor et al., 2010; Grewen and Light, 2011; Carson et al., 2014), the neural mechanisms underlying these effects in humans have not yet been investigated. The present study provides the first clear evidence that plasma AVP levels are associated with anxiogenic effects (i.e., increased amygdala activation during the processing of negative stimuli) in men, but not in women. Importantly, our findings indicate that sex modulates the anxiogenic effects of AVP, which is consistent with previous behavioral studies (Taylor et al., 2010; Brunnlieb et al., 2013; Lee et al., 2013). These results suggest that the neural mechanisms underlying the anxiogenic effects of AVP involve the amygdala, and indicate that AVP may serve as a valid biological marker for the clinical assessment of individual differences pertaining to anxiogenic effects in men.

Strong effects of AVP on amygdala functioning may lead to higher baseline levels of plasma AVP in men relative to women; this asymmetry may be responsible for the fact that significant effects of plasma AVP on amygdala activation are found only in men (Neumann and Landgraf, 2012), although the sex-based difference remains unresolved due to conflicting evidence and an unclear mechanism (Meyer-Lindenberg et al., 2011). AVP neurons project to the amygdala, where AVP receptors are locally expressed at higher levels in males than they are in females (Bamshad et al., 1993; Wang and Vries, 1995). In addition, AVP release within the amygdala has been observed in response to stressful emotional experiences in male rats (Ebner et al., 1999; Bosch and Neumann, 2010), and this release is greater in animals with stronger anxiety behaviors (Bosch and Neumann, 2010). The amygdala also projects to other brain regions, such as the paraventricular nucleus, the bed nucleus of the stria terminalis, and the hypothalamus, all of which exhibit elevated AVP mRNA expression levels (Ma et al., 1997; Wotjak et al., 2001) and are considered to be, along with the amygdala, critically involved in peripheral and central AVP communication. Among these regions, the magnocellular AVP neurons of the paraventricular nucleus communicate with the limbic region, including the amygdala, and ultimately project to the posterior pituitary (Neumann and Landgraf, 2012). This body of evidence pertaining to systemic AVP circulation provides a partial explanation of the mechanism underlying the synergistic nature of peripheral and central AVP processing in men.

We observed male-specific anxiogenic effects of AVP, which are consistent with proposed synergistic mechanisms involving testosterone and AVP in male subjects in animal studies, as described below. The amplifying effects of male gonadal hormones on the influence of AVP on amygdala functioning may result in stronger brain-peripheral AVP communication in men than in women. AVP expression in the amygdala is known to be greatly influenced by gonadal steroids. In rats, testosterone directly acts on AVP-containing cells in the amygdala (de Vries, 2008), and after receiving injections of testosterone, male rats have greater numbers of AVP mRNA-containing cells in the amygdala than do female rats (Urban et al., 1991; De Vries et al., 1994). Testosterone also influences AVP levels in brain regions such as the bed nucleus of the stria terminalis and the hypothalamus, where AVP projections are prominent, and coordinated reactions between these regions and the amygdala have been detected after affective experiences (Albers, 2012). Importantly, testosterone administration has been shown to strengthen AVP fiber networks in the amygdala in male rats (De Vries and Al-Shamma, 1990). Moreover, previous neuroimaging studies have suggested that in men, higher levels of testosterone predicted elevated activation in the amygdala during the processing of threatening stimuli (Manuck et al., 2010). Together with these previous findings regarding the effects of testosterone on AVP in the amygdala, our results would indicate that the observed relationship between peripheral AVP levels and amygdala activation in men may be partly influenced by the effects of testosterone on AVP functioning.

In the present study, endogenous AVP had no effect on the subjective experiences reported by participants while processing negative stimuli. Assessments based on brain measurements (e.g., BOLD signals) may differ among self-reports and peripheral biomarkers. For example, a previous study observed a relationship between brain activation and peripheral biomarkers in the absence of significant associations between self-reports of emotions and peripheral biomarkers (Gross and Levenson, 1993). In addition, intranasal AVP administration was shown to increase amygdala-medial prefrontal cortex activation during the processing of negative stimuli without exerting a significant effect on subjective reports of emotion (Zink et al., 2010). Together with the results of previous studies, our current findings indicate that brain measurements of emotional responses are often more accurately reflective of measured plasma AVP levels than are subjects' emotional self-reports.

Some limitations of the study are worth mentioning in light of our interpretation of the results. First, the sample size was relatively small. Although our sample size represents a relatively large fMRI study, previous studies of plasma neuropeptides have used larger samples (*N* = 473) (Weisman et al., 2013). Second, although our data demonstrated a correlation between amygdala activity and peripheral AVP levels in men, we cannot be sure that the portion of the amygdala we investigated in our study included the region with the highest concentration of OT and AVP receptors. Third, we used increases and decreases in amygdala activation as indices of anxiogenic and anxiolytic effects, respectively. Although our subtraction analysis (aversive pictures minus neutral pictures) yielded robust evidence of activation in the amygdala, resulting in significantly higher levels of activation during the viewing of aversive images relative to neutral ones, we did not find a relationship between subjective reports of chronic negative affect (i.e., trait anxiety) and amygdala activation. This may be because our subjects generally came from a healthy population and did not have particularly high levels of anxiety (mean ± SE, 45.796 ± 8.677 for whole sample; 43.16 ± 6.459 for male, 48.542 ± 9.908 for female). Hence, AVP values may be a more sensitive indicator of amygdala activation than chronic negative affect in healthy populations. For example, a previous study failed to show changes in anxiety scores in healthy men following administration of AVP, but did report increased amygdala-medial prefrontal cortex connectivity in these participants during negative emotional processing (Zink et al., 2010). Future studies are needed to address these issues, including studies that recruit clinical patients or individuals with higher anxiety scores. Fourth, although stage in the menstrual cycle was assessed at the time of blood sampling and was controlled for in our analyses, we did not obtain information pertaining to the menstrual cycle on the day of the MRI, which may have partially distorted the results obtained from our female participants. Fifth, there is considerable controversy regarding the relationship between peripheral levels of these neuropeptides and their central availability. A crucial issue is whether plasma levels of these hormones reflect these brain functions (Meyer-Lindenberg et al., 2011). The relation between central and plasma oxytocin is not completely understood. A further animal study on this issue would be of value to the field. Sixth, the present paper reports mean OT levels that are double the upper threshold of the expected range between 1 and 10 pg/mL (McCullough et al., 2013). We performed extraction procedures prior to conducting the OT assay following the paper's suggestion (McCullough et al., 2013). Even so, the measurements might contain multiple immunoreactive products that are erroneously tagged as OT (McCullough et al., 2013). Some of these non-OT immunoreactive molecules may unexpectedly amplify mean OT levels, which may lead to non-significant results of the relationships between OT and the amygdala activation. Finally, we did not measure plasma OT and AVP on the day of the MRI and we cannot perfectly guarantee the stability of OT and AVP between the time of blood sampling and MRI scanning, leading to some uncertainty about the levels of these neuropeptides on the day of MRI testing; however, our assay supports the day-to-day stability of plasma samples.

To our knowledge, this is the first study to investigate the underlying neural signatures of the anxiolytic effects of OT and the anxiogenic effects of AVP using plasma, and to investigate sex-based modulations of these effects. Our findings showed anxiogenic effects of plasma AVP as reflected in the amygdala activity of men, but not of women. This may reflect more robust peripheral and central AVP communication in men relative to women, possibly influenced by the effects of testosterone on AVP functioning. Our results indicate the potential validity and usefulness of AVP plasma level as an index of individual differences pertaining to anxiogenic effects and as a clinically relevant marker.

## Author contributions

Designed research: KM, MS, HT, and RK. Performed research: KM, HT, YK, SN, and RY. Analyzed data: KM. Drafting of manuscript: KM and MS. Critical revision: KM, MS, HT, YK, SN, RY, and RK.

### Conflict of interest statement

The authors declare that the research was conducted in the absence of any commercial or financial relationships that could be construed as a potential conflict of interest. The reviewer EP and handling Editor declared their shared affiliation, and the handling Editor states that the process nevertheless met the standards of a fair and objective review.
