# Supplementary material for: Are Plasma Oxytocin and Vasopressin Levels Reflective of Amygdala Activation during the Processing of Negative Emotions? A Preliminary Study
Source: Front Psychol. 2016 Apr 8;7:480. doi: 10.3389/fpsyg.2016.00480 (PMC4824786; doi:10.3389/fpsyg.2016.00480)

SUPPLEMENTARY APPENDIX

Figure S1. Significant regions of activation during emotion generation (Look Negative–Look Neutral). These regions survived the cluster-based correction for multiple comparisons. Coordinates are shown in MNI space. Table S1 lists the cluster labels, peak coordinates, T-values, and cluster sizes.

Table S1. Activation results

Regions	x	y	z	T	Cluster size	P
**Emotion generation (Look Negative - Look Neutral)**						
Inferior occipital gyrus	-42	-76	-11	13.84	3439	<0.001
Inferior Frontal Gyrus	45	11	37	7.28	652	<0.001
Superior Frontal Gyrus	3	8	58	6.88	517	<0.001
Middle Frontal Gyrus	-36	-1	37	5.58	415	<0.001
Brainstem	3	-34	-8	5.21	203	0.001
Amygdala	-27	-4	-20	4.89	75	0.043

Table S1. Significant regions of activation during emotion generation (Look Negative–Look Neutral). These regions survived the cluster-based correction for multiple comparisons. Coordinates are shown in MNI space.

Figure S1.

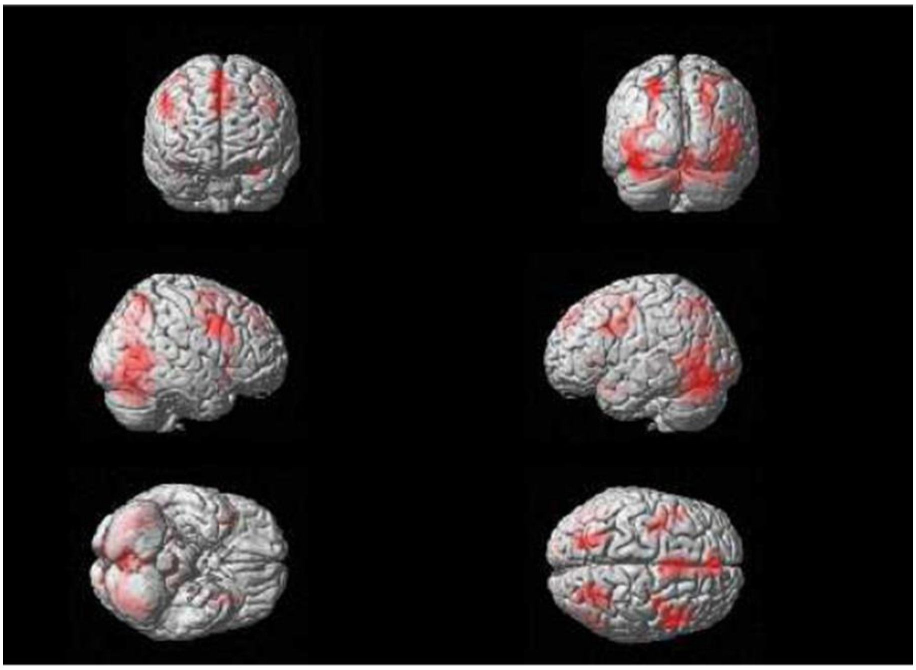

Supplement: Supplementary file 1 [file DataSheet1.pdf]
